# Causal association between family health, perceived relationship quality components, and attitudes toward childbearing in Iranian women: A WHO model analysis

**DOI:** 10.1002/brb3.3625

**Published:** 2024-07-10

**Authors:** Atefeh Taheri, Sara Esmaelzadeh Saeieh, Mostafa Qorbani, Farima Mohamadi, Danial Mahmoodi, Zohreh Mahmoodi

**Affiliations:** ^1^ Student Research Committee Alborz University of Medical Sciences Karaj Iran; ^2^ Social Determinants of Health Research Center Alborz University of Medical Sciences Karaj Iran; ^3^ Non‐Communicable Diseases Research Center Alborz University of Medical Sciences: Endocrinology and Metabolism Research Center, Endocrinology and Metabolism Clinical Sciences Institute Tehran University of Medical Sciences Karaj Iran; ^4^ Social Determinants of Health Research Center Shahid Beheshti University of Medical Sciences Tehran Iran; ^5^ Student Santa Rosa Junior College Santa Rosa California USA

**Keywords:** attitude, childbearing, family health, model, WHO

## Abstract

**Background:**

Given the unprecedented global decline in fertility as a major demographic development in recent years, the present study was conducted to determine Causal association Between Family Health, Perceived Relationship Quality Components, and Attitudes toward Childbearing in Iranian Women: A WHO Model Analysis

**Methods:**

In 2023, this descriptive study recruited 400 married women presenting to selected comprehensive health centers affiliated to Alborz University of Medical Sciences, Karaj, Iran. The data were collected through multistage stratified cluster sampling and using a socioeconomic status questionnaire (Ghodratnama), the Perceived Relationship Quality Components (PRQC) scale, the family‐of‐origin scale (FOS), the attitudes toward fertility and childbearing scale (AFCS) and a demographic checklist were analyzed in SPSS 25 and LISREL 8.8.

**Results:**

According to the path analysis, family health exerted the most significant and positive causal effect on attitudes to childbearing directly through one path (*B* = 0.334) and relationship quality (*B* = 0.698) and duration of married life (*B* = 0.387) both directly and indirectly. The number of children (*B* = –0.057), however, exerted the most significant and negative causal effect on attitudes to childbearing through both paths.

**Conclusions:**

The present findings suggested the significant effects of family health and relationship quality on attitudes toward childbearing. It is therefore recommended that these variables be screened in comprehensive health centers, the associated limitations and problems be identified and appropriate training and counseling solutions be provided by health specialists.

## Graphical abstract

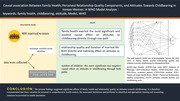

## 1 BACKGROUND

In the 1990s, the total fertility rate began declining to sub‐replacement levels across Europe and even dropped to below 1.3 throughout the Eastern Bloc (Ciritel et al., 2019). In recent decades, the total fertility rate in Iran has decreased from 6.3 to 1.29, suggesting the highest drop compared to other Asian countries (Gubhaju, 2008; Kaboudi et al., 2013). The current fertility rate (2024) for Iran is 2.081 births per woman, a 0.48% decline from 2023 (World Bank, 2024)

The difference in the path of decline in a country is mainly explained by postponing pregnancy. Today, the principle and timing of childbearing are questioned by many people (Rijken & Knijn, 2009). Continuously decreasing fertility gradually alters the age structure of a population from young to old. The consequences of this replacement include numerous national production and economic problems, growing social damage, communication disorders among children in small families, psychological disorders in future generations and excessive healthcare costs for older adults (AbbasiShavazi et al., 2002; Eshaghi et al., 2014; Ghasemi, 2018; Zanjani, 2011).

The values and general health of a family as its biopsychosocial capacity to rear and satisfy the needs of its members (Lima Rodríguez et al., 2012) constitute fundamental components of a social system and affect the inclination and attitudes toward childbearing (Kaveh Firouz et al., 2021). A healthy family is characterized by marital satisfaction, meeting the needs of the members, marital intimacy, and marital cohesion (Panahi & Zarean, 2013).

Qualitatively investigating marital relationship plays a key role in evaluating the overall quality of communication and family health in communities (Paleari et al., 2005). Research suggests positive relationships between the health of the family of origin and marital satisfaction (Salahian et al., 2010). The dimensions of the quality of marital relationship include couples' communication, marital compatibility, satisfaction, happiness, cohesion, and commitment (Paleari et al., 2005). Findings indicate average levels of relationship quality maximize childbearing rates (Rijken & Thomson, 2011).

The structural determinants of health (SDH) have increasingly attracted the attention of the public health community. The WHO model of the SDH categorizes the factors as two groups: (1) sociostructural determinants forming the social class and including education, income, gender and ethnicity (race). The left column comprising factors such as the government, macroeconomic policies, social policies, public policies, culture, and social values constitutes an effective structure in health through socioeconomic status. (2) Intermediary social determinants, including financial conditions such as lifestyle, occupation, and access to food, behavioral and biological factors, psychosocial factors and the health system. These factors affect one another as well as health (Mahmoodi et al., 2013; Solar & Irwin, 2010).

Braveman and Gottlieb (2014) found that the SDH can be directly and indirectly associated with the individual health outcomes such as attitudes toward childbearing. Jalali Aria et al. (2023) reported living conditions and economic instability as the most frequently mentioned barriers by participants. Structural determinant factors, such as undesirable social and economic uncertainty, mostly affect women's decisions about childbearing (Jalali Aria et al., 2023). Deatrick (2017) and Mtenga (2016) found family health and marital relationship quality to lie in the category of the intermediary social determinants of health. Khedmat et al. (2022) mentioned couple's interactions (satisfactory relationships, collaboration, and cooperation) as an important category for childbearing in Iran; in other words, family health has a key role.

Social problems such as declining tendency toward childbearing should be seriously addressed in Iran (Hanson et al., 2011). The relationships of the cited factors with attitudes toward childbearing have been addressed in literature. To the best of the authors’ knowledge, the interrelationships of these factors based on the WHO model have not yet been investigated. Given the key role of childbearing in the rejuvenation of populations, the present research was performed based on the WHO model to examine the relationships of family health and Perceived Relationship Quality Components with attitudes toward childbearing in Iranian women.

We aimed to answer these questions:

1. What is the effect of family health and Perceived Relationship Quality Components (direct/indirect) on attitude to childbearing?
2. What is the effect of Perceived Relationship Quality Components (direct/indirect) on family health?
3. What is the effect of demographic factors (age, education,) on family health, Perceived Relationship Quality Components, and attitude to childbearing?

## 2 METHODS

### 2.1 Study design and participants

This cross‐sectional descriptive‐analytical study was conducted in 2023 (4 February −10 June) by recruiting married women presenting to the selected comprehensive health centers affiliated to Alborz University of Medical Sciences, Karaj, Iran. Karaj is the capital of Alborz Province, the 4th most populous city of Iran and the 22nd most populous metropolis in the Middle East. Maximum demographic diversity was observed in selecting the health centers based on the recommendations of the Health Deputy of Alborz University of Medical Sciences and the data of the Integrated Health System of Iran, locally known as SIB. SIB is the most commonly used information system for recording public health services provided to the Iranian population (Bitaraf et al., 2022).

The sample size calculated as 350 based on the following formula and a study on family health and its effective social factors, 𝜷 = 𝟎.𝟐, 𝜶 = 𝟎.𝟎𝟓, 𝒄𝒐𝒓𝒓𝒆𝒍𝒂𝒕𝒊𝒐𝒏 = 𝟎.𝟏𝟓 (Panahi & Zarean, 2013) was ultimately considered 400 with a dropout rate of 15%.

$$N={\left[\left(Z_{\alpha }+Z_{\beta }\right)/C\right]}^{2}+3,$$

$$C=0.5\times \ln\;\left[\left(1+r\right)/\left(1-r\right)\right].$$

#### 2.1.1 Inclusion criteria

Married women (15–49 years), Iranian nationality, resident in Karaj city, minimum reading and writing literacy, written consent to participate in the study, and no physical or mental illness (registered in the system by self‐reporting).

#### 2.1.2 Exclusion criteria

Infertile women, pregnant women or in the period of exclusive breastfeeding, incomplete answers to the questionnaire questions (failure to answer at least 10% of the questionnaire items).

### 2.2 Data collection

The data were collected using a demographic checklist, the socioeconomic status questionnaire (Ghodratnama), the PRQC, the FOS, and the AFCS.

#### 2.2.1 Demographic checklist

This checklist comprised the individual characteristics of the study subjects and included age, husband's age, ethnicity, husband's ethnicity, age at marriage, husband's age at marriage, duration of married life, number of daughters and sons, gravidity, parity, number of miscarriages, number of stillbirths, education level, husband's education level, occupation status, and husband's occupation status.

#### 2.2.2 Socioeconomic status questionnaire

The socioeconomic status questionnaire developed by Ghodratnama in 2013 with five main items and six demographic items was used to evaluate four dimensions of the socioeconomic status, that is, income level, economic class, education and housing status. The items were scored on a five‐point scale ranging from 1: very low to 5: very high. Eslami et al. (2014) confirmed the face and content validity of this questionnaire in Iran. They also confirmed its reliability by calculating a Cronbach's alpha of 0.83 (2013).

#### 2.2.3 Attitudes toward Fertility and Childbearing Scale (AFCS)

The AFCS developed by Söderberg et al. (2013) was used to evaluate women's attitudes toward fertility and childbearing. The 27‐item English version of this scale comprises three subscales, that is, importance of fertility for the future, childbearing as an obstacle at present and social identity (Söderberg et al., 2013). Baezzat et al. psychometrically analyzed and normalized this scale in Iran (2016). In the 23‐item Persian version of this scale, the child as the pillar of life constitutes the first factor, the child as an obstacle the second, postponing fertility the third, and fertility requiring the fulfillment of prerequisites the fourth. The reliability of this scale was confirmed by calculating a Cronbach's alpha of 0.792. The items were scored on a 5‐point Likert scale (Baezzat et al., 2017).

#### 2.2.4 Family‐of‐Origin Health Scale (FOS)

The 40‐item FOS developed by Hovestadt et al. (1985) was used to measure the respondent's perception of the health level of their family of origin in two dimensions of autonomy and intimacy as key concepts. The items were scored on a 5‐point scale ranging from 1 to 5 (Hovestadt et al., 1985). Kerami (2011) investigated the validity and reliability of this scale in Iran (2009). The construct validity of this questionnaire was confirmed using factor analysis and its reliability by calculating a Cronbach's alpha of 0.86.

#### 2.2.5 Perceived Relationship Quality Components (PRQC)

The 18‐item PRQC developed by Fletcher et al. (2000) measures six dimensions, that is, satisfaction, passion, commitment, intimacy, trust, and love. The items are scored on a 7‐point Likert scale defined as 1: never to 7: absolutely (Fletcher et al., 2000). Several Iranian researchers have investigated the validity and reliability of the PRQC. Rezapour Mirsaleh et al. (2016) confirmed its reliability by calculating a Cronbach's alpha of 0.87.

### 2.3 Procedures

This study began after receiving the necessary permission and approval of the Ethics Committee of Alborz University of Medical Sciences. Multistage stratified cluster sampling was performed by initially dividing Karaj into eastern and western districts as per the municipal division. A list of comprehensive health centers affiliated to the university in each area was then received from the Health Deputy of the university. Afterward, five centers were randomly selected from each district according to the number of centers and population diversity of the area. The required sample size associated with each center was also determined based on the number of its clients. In the next step, the researcher presented to the selected centers, identified eligible candidates by their demographic information and data from SIB (Iran's integrated health system information) and randomly selected some of them. After briefing the selected candidates on the study objectives, those willing to participate in the study were assured of the confidentiality of their information and asked to sign written informed consent forms. The questionnaires were then distributed among the participants in an appropriate setting. The researcher was present to respond to potential questions and ambiguities. The participants who failed to complete the questionnaires in one session were asked to provide their contact details to ensure they would deliver the completed questionnaires within two weeks. The data collected were analyzed in SPSS 25 and LISREL 8.8.

### 2.4 Statistical analysis

**FIGURE 1:**
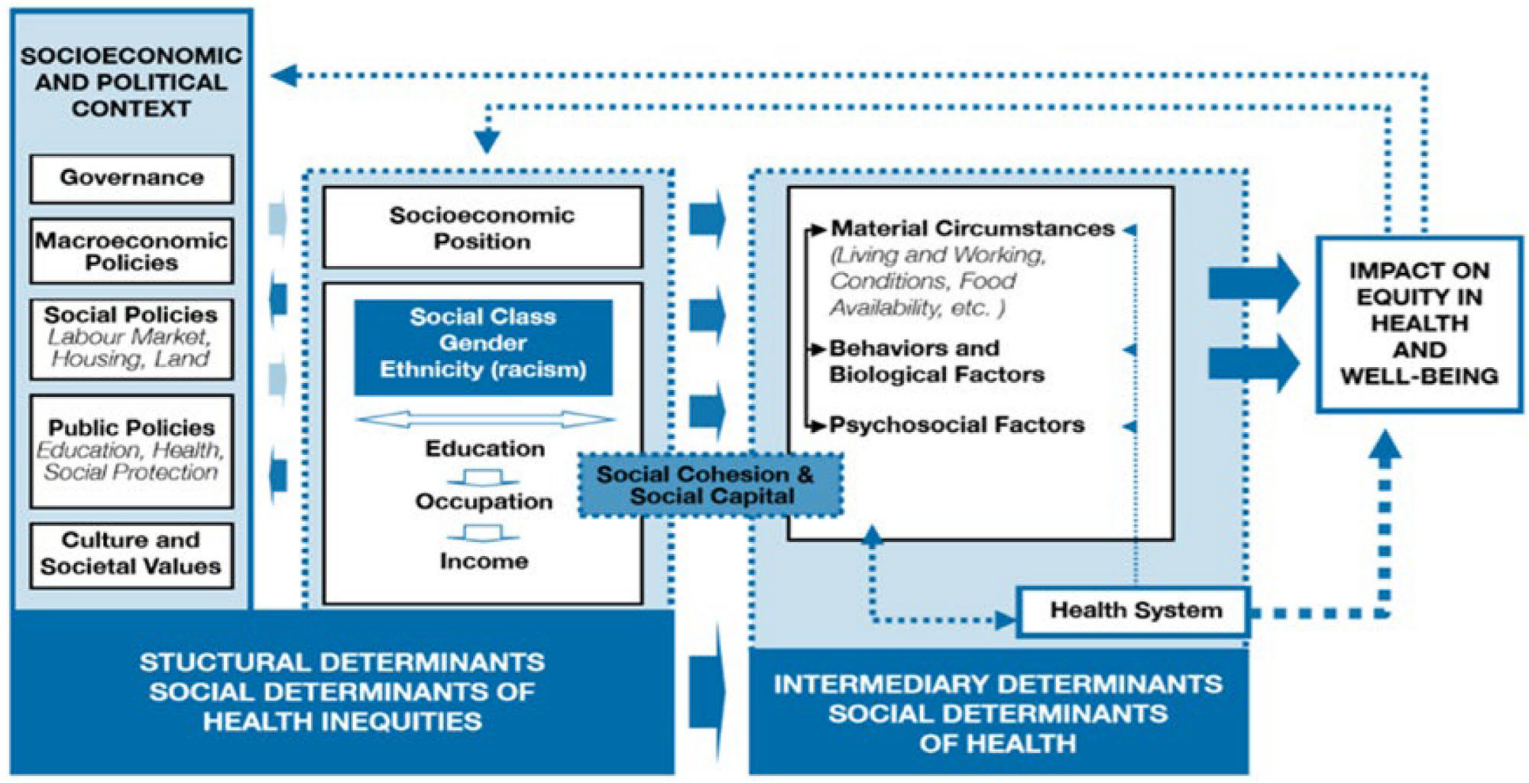
The conceptual framework proposed by the WHO Commission on Social Determinants of Health (Solar & Irwin, 2010).

This study employed the WHO model to investigate the fit of a hybrid model of the relationships of family health and perceived quality of relationship components with attitudes toward childbearing (Figure 1). The distribution normality of the quantitative variables was examined with the Kolmogorov–Smirnov test. Path analysis, a generalization of conventional regression, can reveal the direct and indirect effect of variables in addition to their effect on one another, and hence help to logically interpret the observed relationships and correlations. The data were analyzed in SPSS‐25 (SPSS, 2013) and LISREL 8.8 (Jöreskog & Sörbom, 1996). The correlation and path analysis results were respectively expressed as the Pearson's correlation and beta with a significance level of *T* ≥ 1.96. Path analysis is considered a causal modeling technique; it can be performed with either cross‐sectional or longitudinal data (Plichta et al., 2013).

### 2.5 Variables

All variables in a path model can be described as either endogenous or exogenous. Dependent variables are always endogenous, but some independent (or predictor) variables can be endogenous if influenced by other independent variables in the model (Plichta et al., 2013). In this study, exogenous variables were women's and men's age, women's and men's education, duration of marriage, socioeconomic statues, number of children, and the endogenous variable were attitudes toward childbearing. Family health was exogenous for attitudes toward childbearing and endogenous for Perceived Relationship Quality Components. Perceived Relationship Quality Components were exogenous for Family health and attitudes toward childbearing and endogenous for other variables.

## 3 RESULTS

The present study investigated the data of 400 married women presenting to the selected comprehensive health centers affiliated to Alborz University of Medical Sciences. The mean age of the women was obtained as 34.7 ± 6.3 years and their mean education level as 13.1 ± 3.47. A majority of them (69%) were housewives and belonged to the Persian ethnicity (50%). According to Table 1, the mean score of family health was obtained as 119.6 ± 7.8, the mean score of Perceived Relationship Quality Components as 108.5 ± 14.5 and the total mean score of attitudes toward childbearing as 80.7 ± 9.1 (Table 1).

**TABLE 1:** The sociodemographic characteristics of the participants.

Quantitative						
Variables			Mean ± SD	Variables		Mean ± SD
Women's age [year]			34.7 ± 6.3	Age husband [year]		38.7 ± 7.9
Women's education (year)			13.11 ± 3.44	Men Education (year)		13.12 ± 3.43
Duration of marriage			11.6 ± 7.9	Socioeconomic status		11.2 ± 3.9
Family health		Independence	62.1 ± 4.7	Perceived Relationship Quality Components	Satisfaction	17.3 ± 3.7
					Sexual excitement	16.7 ± 3.4
		Intimacy	57.7 ± 4.6		Commitment	19.3 ± 2.2
					Intimacy	18 ± 3
		Total	119.6 ± 7.8		Confidence	18.5 ± 2.9
					Love	18.9 ± 2.9
					Total	108.8 ± 14.5
Child‐bearing total			80.7 ± 9.1			
Qualitative						
Variables			*f* (%)	Variables		*f* (%)
Women's ethnicity	Fars		200(50)	Men's ethnicity	Fars	191(47.8)
	Lor		41(10.3)		Lor	49(12.3)
	Kord		23(5.8)		Kord	28(7)
	Tork		121(30.3)		Tork	117(29.3)
	Others		15(3.8)		Others	15(3.8)

The Pearson's correlation analysis suggested duration of married life, number of children, socioeconomic status, family health and quality of marital relationship were significantly correlated with attitudes toward childbearing. The most significant positive correlations with attitudes to childbearing were observed in number of children (*r* = 0.08) and family health (*r* = 0.03) (Table 2).

**TABLE 2:** Correlation matrix between total family health score, quality of marital relationship and attitude toward childbearing in married women presenting to comprehensive health centers of Alborz University of Medical Sciences. Sample size = 400 people.

		1	2	3	4	5	6	7	8	9	10
1	Women's age	1									
2	Men's age	0.89**	1								
3	Duration of marriage	0.75**	0.81**	1							
4	Woman's eeducation	–0.15**	–0.18**	–0.30**	1						
5	Men's eeducation	–0.13**	–0.17**	–0.24**	0.68**	1					
6	Number of children	0.43**	0.44**	0.62**	–0.28**	–0.24**	1				
7	Socioeconomic status	0.01	0.003	0.003	0.33**	–0.37	–0.04	1			
8	Perceived Relationship Quality Components	–0.24*	–0.17**	–0.14**	0.08	0.15**	–0.20**	0.11*	1		
9	Family health	0.02	0.05	0.007	0.04	0.05	–0.09*	0.11*	–0.05	1	
10	Attitudes toward childbearing	–0.004	–0.01	0.01**	0.19	0.17	0.08*	0.016**	0.005*	0.03*	1

^**^ *p* = < .01.

^*^ *p* = < .05.

**FIGURE 2:**
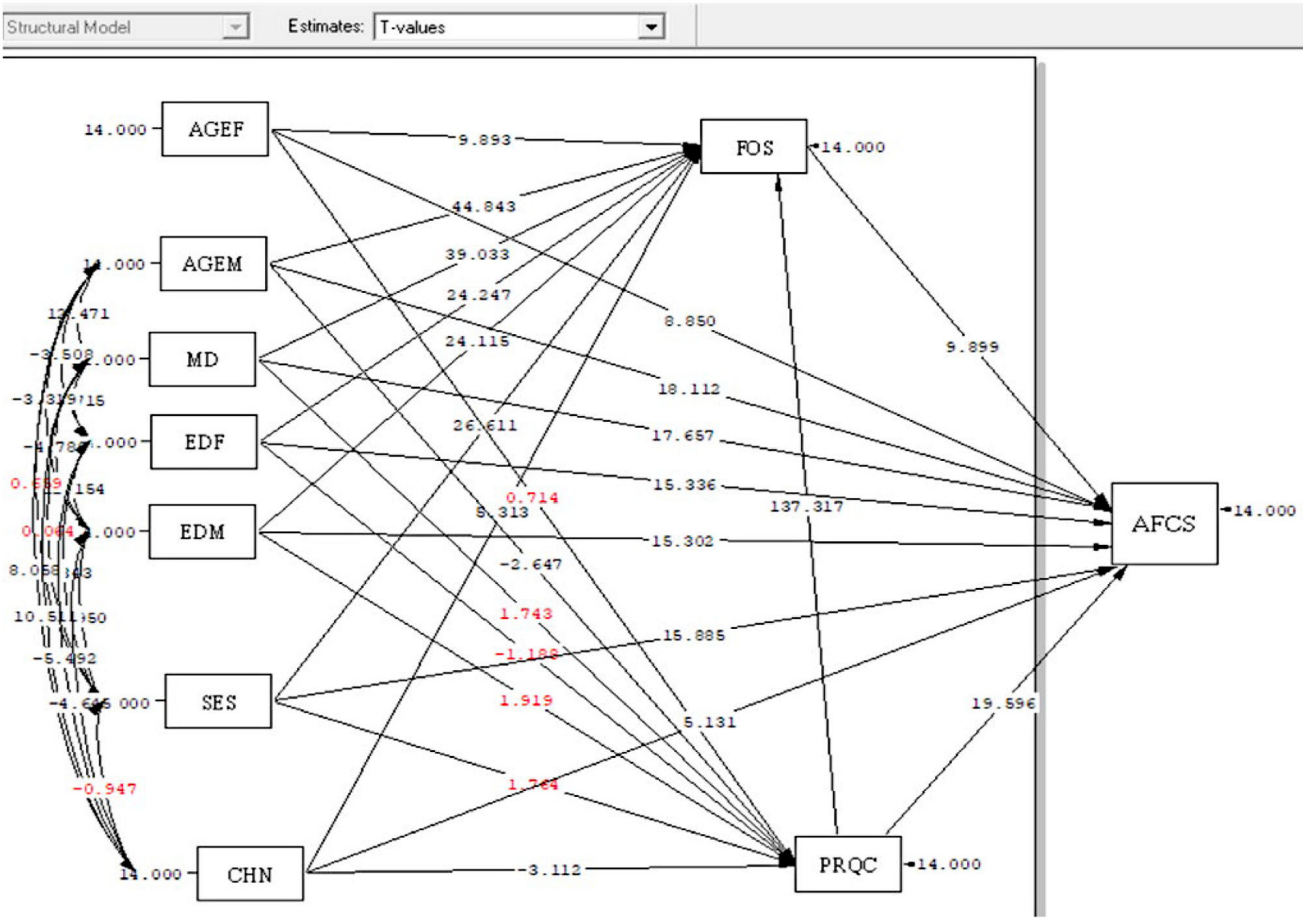
The path analysis model test of demographic factors, the total family health score, marital relationship quality and attitudes toward childbearing by T‐Value. AGEF = age female, AGEM = age male, MD = duration of marriage, EDF = education female, EDM = education men, SES = socioeconomic statues, CHN = child number, FOS = Family‐of‐Origin Health Scale, PRQC = Perceived Relationship Quality Components, AFCS = attitudes toward fertility and childbearing scale.

**FIGURE 3:**
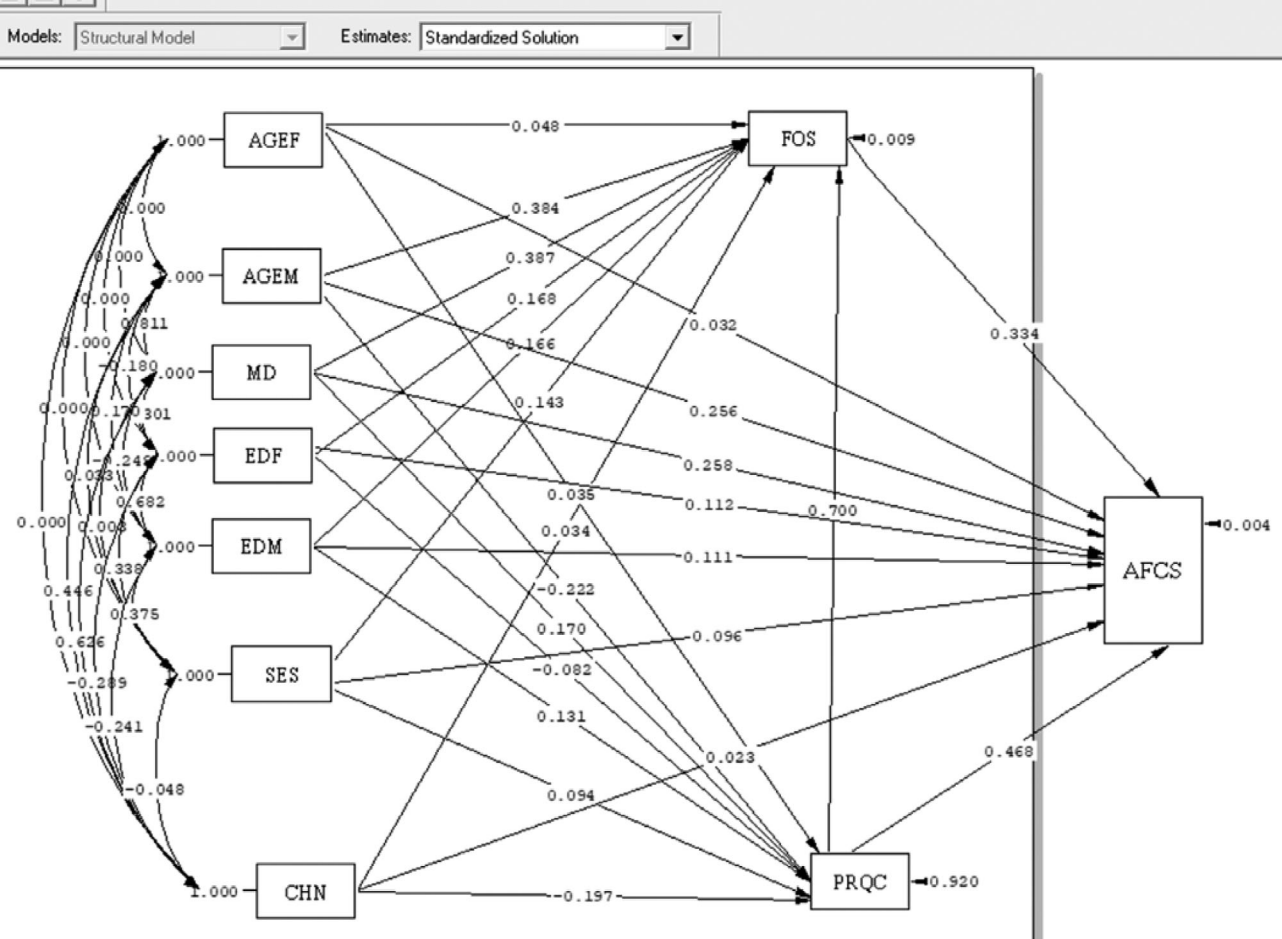
The path analysis model test of demographic factors, the total score of family health, marital relationship quality and attitudes toward childbearing by standardized Beta. AGEF = age female, AGEM = age male, MD = duration of Marriage, EDF = education female, EDM = education men, SES = socioeconomic statues, CHN = child number, FOS = Family‐of‐Origin Health Scale, PRQC = Perceived Relationship Quality Components, AFCS = attitudes toward fertility and childbearing scale.

The path analysis performed by investigating significant paths (*T* ≥ 1.96) (Figure 2). To answer the questions one and three, the result of analysis show that family health was the only variable that directly exerted positive causal effects on attitudes toward childbearing along a single path (*B* = 0.334). In other words, the higher the score of family health, the higher the score of attitudes toward childbearing. The other variables were significantly related to attitudes toward childbearing along both direct and indirect paths. Perceived Relationship Quality (*B* = 0.698) and duration of married life (*B* = 0.387) respectively exerted the most positive causal effect and number of children (*B* = −0.057) the most negative effect. In fact, the higher the quality of marital relationship and the longer the marriage, the higher the attitudes toward childbearing. Higher numbers of children, however, lowered these attitudes. The coefficient of determination obtained as *R* ^2 ^= 0.99 in the preset study model demonstrated the very good adaptation of the theoretical model to reality (Table 3, Figure 3). To answer the second question, the result of path analysis showed that Perceived Relationship Quality had a positive causal effects on family health (*B* = 0.7). In other words, the higher the score of Perceived Relationship Quality, the higher the score of family health. (Figure 3)

**TABLE 3:** Direct and indirect effects of individual factors, total family health score, quality of marital relationship with attitude toward childbearing, sample size = 400 people.

	Standard				
Variable	Direct effect	Indirect effect	Total effect	*T*‐value direct effect	*R* ^2^
Women's age		0.16*	0.192*	8.85	0.99
Men's age	0.256*	0.01*	0.266*	18.11	
Duration of marriage	0.258*	0.12*	0.378*	17.65	
Women's eeducation	0.112*	0.05*	0.162*	15.33	
Men's eeducation	0.111*	0.05*	0.161*	15.30	
Number of children	0.023*	–0.08*	–0.057*	5.13	
Socioeconomic status	0.096*	0.04*	0.136*	15.88	
Perceived Relationship Quality Components	0.468*	0.23*	0.698*	19.59	
Family health	0.334*	_	0.334*	9.89	

The model fit indices demonstrated the goodness of fit of the model and the rationality of the adjusted relationships between the variables based on the conceptual model. Insignificant differences were therefore observed between the fitted and conceptual model (Table 4).

**TABLE 4:** The fit indices of the correlation model between individual factors, the total family health score, the quality of the marital relationship with the attitude toward childbearing, sample size = 400 people.

	*X* ^2^	df	*X* ^2^/df	NFI	NNFI	CFI	GFI	AGFI	RMSEA
Fit index	10.55	5	2.11	0.99	0.99	0.98	0.99	0.96	0.01
Standard	*X* ^2^/df < 5	>0.90	>0.90	>0.90	>0.90	>0.90	>0.90	<0.05	

## 4 DISCUSSION

According to the results of the path analysis, among the variables with causal relationships with attitudes toward childbearing, family health was directly, positively, and significantly related to these attitudes. Moreover, marital relationship quality (Perceived Relationship Quality Components) was both directly and indirectly through family health significantly and positively related. In other words, increases in the scores of family health and marital relationship quality promoted attitudes toward childbearing. These findings were consistent with those obtained by Panahi and Zarean (2013), Rijken and Thomson (2011), and Slatcher and Schoebi (2017). According to Panahi et al., relationships in a healthy family are established based on mutual respect, where love and intimacy prevail among the members. Roles are predefined, healthy relationships exist among the members, and their satisfaction and mental and moral health are ensured in a healthy family (Panahi & Zarean, 2013). Khedmat et al. (2022) explained the following four categories as more effective on childbearing in Iran: couple's capability, parenting attitudes (paternal and maternal feelings, emotional support, social support, and age role), couple's interactions (satisfactory relationships, collaboration, and cooperation), and childbearing experiences. They indicated that most couples believed that couple's satisfactory relationships affected their childbearing intention.

Empathy is necessary before childbearing. As the fundamental factors associated with childbearing and major components of a social system, the values and general health of a family can be managed to help the community achieve prosperity (Kaveh Firouz et al., 2021). Strengthening the family system plays a key role in promoting the health of the family and community (Simforosh & Ghazi‐Tabatabaee, 2017). According to the conceptual model of family health proposed by Panahi and Zarean (2013), a healthy family is characterized by marital satisfaction, meeting the needs of members, intimacy and marital cohesion and family health is influenced by the quality of marital relationship which, in turn, is affected by numerous factors, including marital satisfaction as a major factor. This variable helps cope with problems and stresses and improve marital mental health (Navabi‐Najad, 2008). According to Slatcher and Schoebi (2017), the positive outcomes of stable and satisfactory marital relationships for family members include psychophysical health and effective growth. These marital relationships pave the way for the growth and health of family members, including children. Research suggests the positive dimension of the quality of married life significantly affects the intention to have a/another child in couples without children or with one child. This dimension was, however, found not to significantly affect families with at least two children. In other words, the higher quality of married life is effective in changing the state of the family from childlessness or having one child (Modiri & Tabatabaie, 2019). Rijken and Thomson (2011) found the moderate quality of marital relationship to maximize the childbearing rate, and women's perception of the quality of the relationship affects the probability of the first birth.The qualitative dimensions of married life can affect demographic variables. The quality of marital relationship influences both childrearing and childbearing (Moshfegh et al., 2022).

In line with the studies by Fallahzadeh et al. (2017), (Mobasheri et al., 2015), and Keshavarzmozafar et al. (2014), the present findings suggested significant causal relationships between duration of married life and attitudes toward childbearing along both direct and indirect paths. Mobasheri et al. (2015) reported significant and direct relationships between the score of attitudes toward childbearing and duration of married life. In other words, the higher the age of the woman and the more prolonged the married life, the higher the number of children. They reported more opportunities to have children in older women (Fallahzadeh et al., 2017). Keshavarzmozafar et al. (2014) also found fertility rates to significantly relate to duration of married life and age at first marriage.

In line with the studies by Bagi et al. (2022) and Moradi and Saffarian (2019), the present research found the number of children to constitute the only variable with the most significant and negative causal relationships with attitudes toward childbearing along both direct and indirect paths.

In fact, attitudes to childbearing decreased with increases in the number of children. Bagi et al. (2022) found the number of children to constitute a major determinant in one's desire to have another child. They found concerns about children's problems and future to be an effective factor in unwillingness to have children. Over 60% of the subjects reported socioeconomic problems as the main obstacle to their willingness to have more children (Bagi et al., 2022). The two opposing attitudes toward childbearing in developing communities reported by Moradi and Saffarian (2019) included considering children as future's workforce and the role of religion in promoting childbearing. On the other hand, economic and technological developments and high costs of childrearing in these communities yield diminishing attitudes toward childbearing. The latter approach appears to dominate according to these results and the study community (Moradi & Saffarian, 2019). In contrast, Taghvaee‐Fard et al. (2020) found increasing numbers of children to raise willingness to have more children. The discrepancy in results can be explained by differences in the instrument used to measure attitudes toward childbearing. These authors employed a researcher‐made questionnaire, whereas the present research utilized the standard AFCS (Söderberg et al., 2013). Differences in the study population might have also contributed to the differences in the results. These authors collected their sample from married women living in Jahrom, the south of Fars Province, Iran, whereas the present study sample was collected from married women in Karaj. Furthermore, cultural and ethical differences between the two provinces might have contributed to the discrepancy.

## 5 LIMITATION

The limitation of the present research is that we used questionnaires to collect and record the data, and the number of questions can affect individual's accuracy. Furthermore, the samples enrolled in this study were not taken from all parts of Karaj city although we tried to enroll a diverse sample.

## 6 CONCLUSION

Based on our findings, family health and the quality of marital relationship are crucial to and effective in attitude toward childbearing. Accordingly, it is recommended to address, strengthen and improve the quality of couple's communication as an appropriate method for improving mental health, life satisfaction and hence, attitude toward childbearing. It is also recommended to examine and screen the health of families in comprehensive health centers and run effective workshops to improve communication skills and intimacy between couples. These recommendations can be adopted for policy‐making and planning related to Iran's family and population.

## 7 POLICY IMPLICATION OF FINDINGS

According to the country's context, a multidimensional program is essential to promote childbearing. Our findings show that family health and Perceived Relationship Quality exert positive causal effects on attitudes toward childbearing; therefore, policymakers have to devise programs for screening and improving family health and relationship to improve childbirth.

## AUTHOR CONTRIBUTIONS

**Atefeh Taheri**: Investigation; writing—original draft; data curation; conceptualization; writing—review and editing. **Sara Esmaelzadeh Saeieh**: Writing—original draft; writing—review and editing; conceptualization. **Mostafa Qorbani**: Writing—review and editing; writing—original draft; investigation; methodology. **Farima Mohamadi**: Formal analysis; data curation; writing—review and editing; writing—original draft. **Danial Mahmoodi**: Writing—original draft; writing—review and editing; software; formal analysis. **Zohreh Mahmoodi**: Conceptualization; investigation; writing—original draft; writing—review and editing; methodology; project administration.

## FUNDING

This research did not receive any specific grant from funding agencies in the public, commercial, or not‐for‐profit sectors

## CONFLICT OF INTEREST STATEMENT

The authors declare that they have no competing interests

### PEER REVIEW

The peer review history for this article is available at https://publons.com/publon/10.1002/brb3.3625.

## CONSENT FOR PUBLICATION

All of the authors have consent for publication

## Data Availability

The data that support the findings of this study are available from the corresponding author upon reasonable request
